# Integrated Assessment of Heavy Metal Contamination in Sediments from a Coastal Industrial Basin, NE China

**DOI:** 10.1371/journal.pone.0039690

**Published:** 2012-06-29

**Authors:** Xiaoyu Li, Lijuan Liu, Yugang Wang, Geping Luo, Xi Chen, Xiaoliang Yang, Bin Gao, Xingyuan He

**Affiliations:** 1 State Key Laboratory of Desert and Oasis Ecology, Xinjiang Institute of Ecology and Geography, Chinese Academy of Sciences, Xinjiang, China; 2 State Key Laboratory of Forest and Soil Ecology, Institute of Applied Ecology, Chinese Academy of Sciences, Liaoning, China; 3 College of Environmental Science and Forestry, State University of New York, Syracuse, New York, United States of America; 4 College of Resources Science and Technology, Beijing Normal University, Beijing, China; Argonne National Laboratory, United States of America

## Abstract

The purpose of this study is to investigate the current status of metal pollution of the sediments from urban-stream, estuary and Jinzhou Bay of the coastal industrial city, NE China. Forty surface sediment samples from river, estuary and bay and one sediment core from Jinzhou bay were collected and analyzed for heavy metal concentrations of Cu, Zn, Pb, Cd, Ni and Mn. The data reveals that there was a remarkable change in the contents of heavy metals among the sampling sediments, and all the mean values of heavy metal concentration were higher than the national guideline values of marine sediment quality of China (GB 18668-2002). This is one of the most polluted of the world’s impacted coastal systems. Both the correlation analyses and geostatistical analyses showed that Cu, Zn, Pb and Cd have a very similar spatial pattern and come from the industrial activities, and the concentration of Mn mainly caused by natural factors. The estuary is the most polluted area with extremely high potential ecological risk; however the contamination decreased with distance seaward of the river estuary. This study clearly highlights the urgent need to make great efforts to control the industrial emission and the exceptionally severe heavy metal pollution in the coastal area, and the immediate measures should be carried out to minimize the rate of contamination, and extent of future pollution problems.

## Introduction

Coastal and estuarine areas are among the most important places for human inhabitants [1]; however, with rapid urbanization and industrialization, heavy metals are continuously carried to the estuarine and coastal sediments from upstream of tributaries [2]–[5]. Heavy metal contamination in sediment could affect the water quality and bioaccumulation of metals in aquatic organisms, resulting in potential long-term implication on human health and ecosystem [6]–[7]. In most circumstances, the major part of the anthropogenic metal load in the sea and seabed sediments has a terrestrial source, from mining and industrial developments along major rivers and estuaries [8]–[10]. The hot spots of heavy metal concentration are often near industrial plants [11]. Heavy metal emissions have been declining in some industrialized countries over the last few decades [12], [13], however, anthropogenic sources have been increasing with rapid industrialization and urbanization in developing countries [14], [15].Heavy metal contaminations in sediment could affect the water quality, the bioassimilation and bioaccumulation of metals in aquatic organisms, resulting in potential long-term affects on human health and ecosystem [16]–[19]. Quantification of the land-derived metal fluxes to the sea is therefore a key factor to ascertain at which extent those inputs can influence the natural biogeochemical processes of the elements in the marine [20], [21]. The spatial distribution of heavy metals in marine sediments is of major importance in determining the pollution history of aquatic systems [22], [23], and is basic information for identifying the possible sources of contamination and to delineate the areas where its concentration exceeds the threshold values and the strategies of site remediation [24]. Therefore, understanding the mechanisms of accumulation and geochemical distribution of heavy metals in sediments is crucial for the management of coastal environment.

China’s rapid growth of the economy since 1979 under the reform policies has been accompanied by considerable environmental side effects [25]. China is one of the largest coastal countries in the world. Booming coastal urban areas are increasingly dumping huge industrial and domestic waste at sea [26]. The elevated metal discharges put strong pressure on China’s costal and estuarine area. The average annual input of metals by major rivers was approximately 30,000 t between 2002 and 2008 [27]. Chinese government indicates that 29,720 km^2^ of offshore areas of China are heavily polluted [28]. “Hot spots” of metal contamination can be found along the coast of China [29], from the north to the south, especially in the industry-developed estuaries, such as the Liaodong Bay [30] and Yangtze River catchment [31] and Xiamen Bay [32]. In 2002, China enforced Marine Sediment Quality (GB 18668-2002) to protect marine environment (CSBTS, 2002). Therefore, Marine Sediment Quality (GB 18668-2002) is used as a general measure of marine sediment contamination in China.

Jinzhou Bay, surrounded by highly industrialized regions, is considered as one of the most contaminated coastal areas in China [33]. China produces the largest amount of zinc (Zn) in the world, which was 1.95 million tons in 2000 and will grow to 14.9 million tons in 2010 [26], [34]. And the largest zinc smelting plant in Asia was located at the coast of Jinzhou bay. From 1951 to 1980, the amount of Zn, Cu, Pb and Cd discharged from Huludao Zinc Smelter to Jinzhou bay reached 33745, 3689, 3525 and 1433 t respectively [35]. Although several heavy metal contamination studies have conducted in Jinzhou Bay area recently, these studies were focused on coastal urban soils [36], river sediments [37] and seawater [38] separately. Few researches take the coastal stream, estuary and bay as a whole unit to assess the heavy metal contamination of coastal industrial area spatially and temporally. Thus, it is necessary to understand the process of heavy metal contamination and to evaluate the potential ecological risks of heavy metals in the coastal stream, estuary and bay integratively.

In recent decades different metal assessment indices applied to sediment environments have been developed. Caeiro et al [9] classified them in three types: contamination indices, background enrichment indices and ecological risk indices. The geo-accumulation index (I_geo_) [39] and the potential ecological risk index (RI) [40] are the most popular methods used to evaluate the ecological risk posed by heavy metals in sediments [41]–[44]. RI method considers the toxic-response of a given substance and the total risk index, and can exhibit the actual pollution condition of seriously polluted sediment [45], [46].

Over the last few decades the study of the sediment cores has shown to be an excellent tool for establishing the effects of anthropogenic and natural processes on depositional environments [44], [47]. Sediment cores can be used to study the pollution history of aquatic ecosystem [48], [49]. Within an individual sediment core, differences in pollutant concentrations at different depths reflect how heavy metal input and accumulation changes over time [50], [51].

The purpose of this study is (1) to quantify and explain the spatial distribution of heavy metal contaminants in modern sediments of Jinzhou bay, NE China; (2) to investigate the natural and anthropogenic processes controlling sediment chemistry; and (3) to identify the potential ecological risks of such heavy metals.

## Materials and Methods

### Study Area

**Figure 1:**
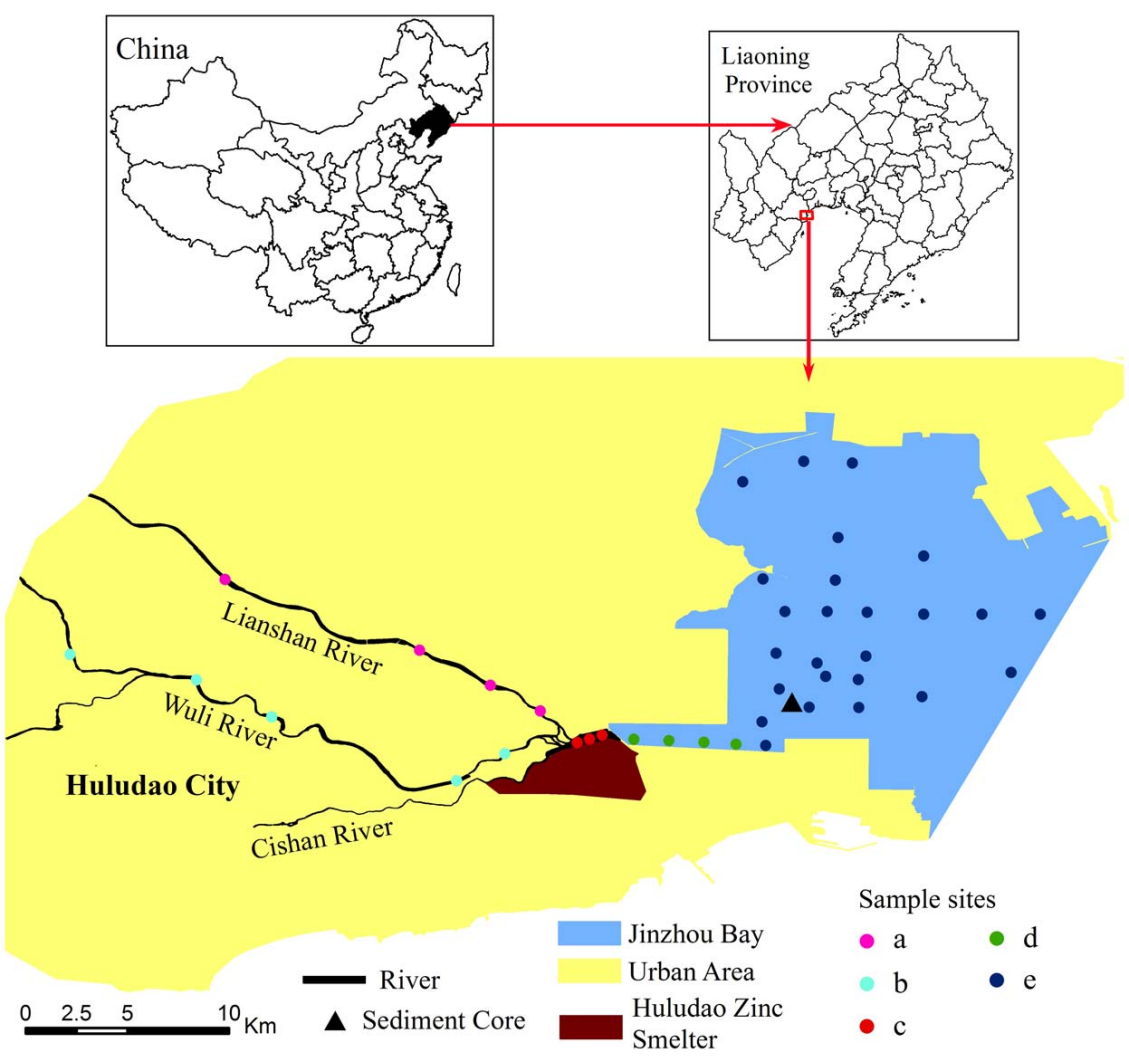
The sampling sites in the study area. (a, Lianshan River upstream of Huludao Zinc Smelter (n = 4); b, Wuli River upstream of Huludao Zinc Smelter (n = 5); c, Converged river Downstream of Huludao Zinc Smelter (n = 3); d, Estuary (n = 4); e, Jinzhou Bay (n = 25)).

This study was carried out in Jinzhou Bay and its coastal city, Huludao City, in Liaoning Province, northeast of China (Fig. 1). Jinzhou Bay is one of the important bays in the northwest of Liaodong Bay at the northwestern bank of China’s Bohai Sea. It is a semi-closed shallow area with an average depth of 3.5 m and an approximate area of 120 km^2^. Huludao city is located at southwestern coast of Jinzhou bay. The city is an important non-ferrous smelting and chemical industry area in northeast China. More than forty different mineral resources have been discovered in the Huludao region, including gold, zinc, molybdenum, lime and manganese. The economy is dominated by some of China’s most important industrial enterprises, such as Asia’s biggest zinc manufacturing operation, the Huludao Zinc Smelter (HZS), the Jinxi Oil Refinery and Jinhua Chemical Engineering, and Huludao’s Massive Shipyard. The Wuli River, Lianshan River and the Cishan River are three main rivers in the city, flowing into Jinzhou Bay. The water, soil and sediment in the city and Jinzhou bay were heavily polluted by industrial activities. Land reclamation from sea by landfill of soils and solid wastes further increase the level of pollution of the sedimentary environments in this area. These anthropogenic activities have created great threat to the public health and the regional biological and geochemical conditions.

No specific permits were required for the described field studies. The studying area is not privately-owned or protected in any way and the field studies did not involve endangered or protected species.

### Sampling and Analysis

Twelve samples of river sediment were collected from the two major rivers (Lianshan river and Wuli river) and and four samples were collected from their estuary of Huludao City, using a stainless steel shovel. Twenty-five of surface sediments (0–5 cm) and one sediment core were collected in Jinzhou Bay using a stainless gravity corer (40 cm length and 5 cm diameter). The sediment core was sectioned at 2 cm intervals, and each fraction (subsamples) was sliced into 50 ml polyethylene centrifuge tubes with the help of PVC spatula. All the samples were collected in October 2009 in one week.

The samples were oven-dried at 45°C for 3 days, and sieved through a 2-mm plastic sieve to remove large debris, gravel-size materials, plant roots and other waste materials, and stored in closed plastic bags until analysis. Soil was digested with a mixture 5∶2∶3 of HNO_3_–HClO_4_–HF. The digested solutions were analyzed via an inductively coupled plasma-atomic emission spectroscopy (ICP-AES; Perkin Elmer Optima 3300 DV). All of the soil samples were analyzed for total concentrations of Cu, Zn, Pb, Ni, Mn and Cd.

### Statistical Analysis

Statistical methods were applied to process the analytical data in terms of its distribution and correlation among the studied parameters. The commercial statistics software package SPSS version 17.0 for Windows was used for statistical analyses in present study. Basic statistical parameters such as mean, median, standard deviation (SD), coefficient of variation (CV), skewness and kurtosis were computed. To identify the relationship among heavy metals in sediments and their possible sources, Pearson’s correlation coefficient analysis were performed.

### Geostatistical Methods

Semivariogram is a basic tool of geostatistics and also the mathematical expectation of the square of regional variable *z*(*x_i_*) and *z*(*x*+*h_i_*) increment, namely the variance of regional variable. Its general form is:
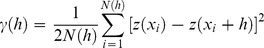
where *r*(*h*) is semivariogram; *h* is step length, namely the spatial interval of sampling points used for the classification to decrease the individual number of spatial distance of various sampling point assemblages; *N(h)* is the logarithm of sampling point when the spacing is *h*; *z*(*x_i_*) and *z*(*x_i_*+*h*) are the values when the variable Z is at the *x_i_* and *x_i_*+*h* positions respectively. The residual sums of squares (RSS), the determining coefficient (R^2^) and F test were used to evaluate the accuracy of the interpolated results.

Kriging, as a geostatistical interpolation method, uses the semivariogram to quantify the spatial variability of regionalized variables, and provides parameters for spatial interpolation. The maps of spatial distribution of heavy metal concentrations were generated by Kriging interpolation with the support of the statistical module of ArcGIS-Geostatistical Analyst.

### Potential Ecological Risk

To assess the effect of multiple metal pollutions in the sediments from the river, estuary and Jinzhou bay, potential ecological Risk Index (RI) was used, which was originally developed by Håkanson [40] and is widely used in ecological risk assessments of heavy metals in sediments. According to this methodology, the potential ecological risk index (RI) is defined as
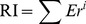
(1)

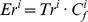
(2)

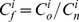
(3)where RI is calculated as the sum of all risk factors for heavy metals in sediments; 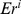 is the monomial potential ecological risk factor; 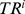 is the toxic-response factor for a given substance (e.g., Cu =  Pb =  Ni = 5, Zn = 1, Cd = 30); 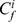, 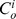 and 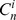 are the contamination factor, the concentration of metals in the sediment and the background reference level, respectively. The background values of Cu, Zn, Pb and Cd are defined as the maximum values of the first category standard of national guideline values of marine sediment quality of China (GB18668-2002) and Ni is defined as the average value of Ni in residual fraction determined, they were 35 mg/kg for Cu, 150 mg/kg for Zn, 60 mg/kg for Pb, 0.5 mg/kg for Cd, and 9 mg/kg for Ni. The concentration of Mn in the sediments showed very weak relationship with the industrial activities, so it was not included in the calculation process of RI.

Still according to Hakanson [40] the following terminology is indicated to be used for the RI value:

RI <150, low ecological risk for the sediment;

150≤ RI <300, moderate ecological risk for the sediment;

300≤ Ri <600, considerable ecological risk for sediment;

RI ≥600, very high ecological risk for the sediment.

## Results and Discussion

### Heavy Metal in the Sediments

Descriptive statistics of heavy metal concentrations of sediments present in rivers of Huludao city, estuary and Jinzhou bay (Fig. 1) are presented in Table 1, 2, 3. As confirmed by the skewness values (Table 1, 3), the concentrations of elements (except Mn) are characterized by large variability, with positively skewed frequency distributions. This is common for heavy metals, because they usually have low concentrations in the environment, so that the presence of a point source of contamination may cause a sharp increase in local concentration, exceeding the thresholds [24].

**Table 1:** Heavy metal concentrations (mg/kg) of River sediments.

		Minimum	Maxinum	Mean	Median	SD	CV%	Skewness	Kurtosis	national guideline values
	a	67.00	186.50	116.50	106.25	50.22	43.11	1.15	2.24	
Cu	b	32.15	85.50	50.61	42.40	24.55	48.51	1.45	1.73	35.00
	c	795.00	2535.00	1533.33	1270.00	899.39	58.66	1.20	–	
	a	471.61	965.31	633.85	549.24	224.08	35.35	1.83	3.51	
Zn	b	153.52	473.93	256.78	199.85	146.71	57.13	1.84	3.50	150.00
	c	1825.96	11010.02	6546.57	6803.73	4597.42	70.23	-.251	–	
	a	62.64	185.10	112.28	100.69	52.34	46.62	1.18	1.76	
Pb	b	40.15	98.47	57.60	45.89	27.47	47.69	1.90	3.66	60.00
	c	417.58	6090.90	2431.09	784.81	3174.79	130.59	1.70	–	
	a	35.69	109.99	57.41	41.98	35.23	61.36	1.94	3.79	
Ni	b	28.28	35.94	31.49	30.885	3.31	10.51	0.92	0.52	–
	c	40.77	87.93	62.58	59.06	23.77	37.98	0.65	–	
	a	520.89	1283.46	812.91	723.65	327.98	40.34	1.47	2.75	
Mn	b	520.21	903.43	710.96	710.11	205.78	28.94	0.004	−5.81	–
	c	337.93	1358.55	915.18	1049.07	523.31	57.18	−1.08	–	
	a	25.53	98.78	53.18	44.21	32.61	61.32	1.29	1.30	
Cd	b	8.04	17.75	11.12	9.35	4.49	40.38	1.81	3.29	0.50
	c	136.73	1019.10	503.51	354.71	459.62	91.28	1.30	–	

a Lianshan River upstream of Huludao Zinc Smelter (n = 4);

b Wuli River upstream of Huludao Zinc Smelter (n = 5);

c Converged river Downstream of Huludao Zinc Smelter (n = 3). The locations of sampling sites are on Fig. 1.

**Table 2:** Heavy metal concentrations (mg/kg) of Estuary sediments.

Distance from HZS to the sampling sites	Cu	Zn	Pb	Ni	Mn	Cd
400 m	1510.00	9304.24	1414.14	59.95	744.13	269.34
1200 m	805.00	3898.09	561.21	64.10	677.46	181.90
2000 m	675.00	2750.57	486.43	48.14	514.07	118.08
2800 m	505.00	3221.59	421.28	53.85	682.69	98.29

The locations of sampling sites are on Fig. 1.

**Table 3:** Heavy metal concentrations (mg/kg) of Jinzhou Bay sediments (n = 25).

	Minimum	Maxinum	Mean	Median	SD	CV%	Skewness	Kurtosis	national guideline values
Cu	24.45	327.50	74.11	51.50	68.54	92.48	2.706	7.964	35.00
Zn	168.06	2506.33	689.39	550.58	568.50	82.46	2.183	4.656	150.00
Pb	29.17	523.45	123.98	89.29	114.70	92.51	2.398	5.934	60.00
Ni	26.29	85.99	43.47	41.41	11.92	27.42	1.835	8.867	–
Mn	445.57	1123.03	750.64	774.00	153.82	20.49	0.311	0.137	–
Cd	7.91	105.31	26.81	20.74	22.23	82.92	2.388	6.155	0.50

The locations of sampling sites are on Fig. 1.

#### Heavy metal in river sediments

Among the concentrations of heavy metal of river sediments (Table 1), the low values are from the sediments sampled before the river flowing by the Huludao Zinc Smelter (HZS), and the highest heavy metal concentrations come from the two sediment samples collected after the river flows by the HZS and accepted the wastewater discharged from HZS. Although the Wuli river is the least contaminated river in Huludao City; however the concentration of Cd exceeded the national guideline values of marine sediment quality of China (GB 18668-2002) by 22 times. In the upstream of HZS the sediment contamination of Lianshan river was higher than that of Wuli river. These data showed many other industrial operations at upper reaches of HZS also contributed great heavy metals to the river sediments. According to historical data, wastes from Jinxi Chemical Factory and Jinxi Petroleum Chemical Factory were discharged into Wuli River directly for nearly 40 years till 2000 and caused heavy contamination of river sediments. At the same time, wastewater from other small industrial plants and residents, and nonpoint pollution from soils with runoff or atmospheric deposition contributed additional pollution sources [52], [53]. This is be confirmed by the previous studies about contamination of urban soils [36], [54] and river sediments [37] in Huludao city.

The mean concentrations of Cu, Zn, Pb and Cd in the sediment of converged river downstream of HZS adjacent to the estuary exceeding the national guideline values of marine sediment quality of China (GB 18668-2002) by 42, 42, 40 and 1006 times respectively. These indicated that the Huludao Zinc Smelter (HZS) is the largest source of heavy metals in river sediment adjacent to the estuary. The annual amount of discharged wastewater from HZS is estimated to be more than 8 million tons directly to the Wuli river [35]. This situation lasted for more than 50 years till the water reuse was realized around year 2000 [28].

#### Heavy metal in the estuary sediments

Four sediment samples were collected along the estuary, and the distance from HZS to the sampling sites increases from 400 m with an 800 m-interval to the Jinzhou Bay (Fig. 1). All the investigated heavy metals clearly showed the same distribution trend. The maximum concentrations of heavy metals declined as the distance increased from HZS (Table 2). The mean concentration of Cu, Zn, Pb and Cd at estuary were exceeded the national guideline values of marine sediment quality of China (GB 18668-2002) by 24, 31, 11 and 332 times respectively.

Contaminant distributions in the Hudson River estuary were identified two types of trends: increasing trend down-estuary dominated by down-estuary sources such as wastewater effluent, and decreasing trend toward bay dominated by upriver sources, where they are removed and diluted downstream along with the sediment transport [55]. The result of this study showed obvious decreasing trend toward bay. This confirmed that the wastewater discharged from HZS and other industrial plants were the main sources of heavy metals in sediment.

#### Heavy metal in Jinzhou Bay sediments

Twenty-five of surface sediments from Jinzhou Bay were tested and the minimum, maxinum and mean values are all located in Table 3. There were remarkable changes in the concentrations of heavy metals in the sediments of Jinzhou bay (Fig. 1). The heavy metal concentration levels are comparable to those with previous studies [56]–[58], especially for Cu, Pb and Zn, Mean concentrations of Cu, Zn, Pb and Cd were as high as 2.1, 4.6, 2.1 and 53.6 times of the national guideline values of marine sediment quality of China (GB 18668-2002). According to the research results from Institute of Marine Environmental protection, State oceanic Administration of China in 1984, the background value of Cu, Zn, Pb and Cd in Jinzhou bay was 10.0, 48.59, 9.0 and 0.29 mg/kg respectively [35]. This clearly demonstrates an anthropogenic contribution and reveals a serious pollution of sediments in Jinzhou bay. For all metals, total concentrations had a great degree of variability, shown by the large coefficients of variation (CV) from 20.49% of Mn to 92.51% of Pb. The elevated coefficients of variations reflected the inhomogeneous distribution of concentrations of discharged heavy metals. Large standard deviations were found in all heavy metals levels. The results of the K–S test (P<0.05) showed that the concentrations of measured metals were all normally distributed.

The comparison of contaminant concentrations observed in this study with those reported for other regions (Table 4) indicates that levels and ranges of variation of our data are similar to those reported from sites with high anthropogenic impact. It was found that the concentrations of Cd measured in this study were greatly higher than other studies except that of Algeciras Bay was a little close to this study. The levels of Zn and Pd in this study were only lower than that of Izmit Bay and Gulf of Naples respectively. The contents of Cu were relatively higher than other study (except Izmit Bay, Bay of Bengal and Hong Kong). All these show that Jinzhou bay was a highly polluted area in the world.

**Table 4:** Mean concentrations (mg/kg) of heavy metals found in Jinzhou bay compared to the reported average concentrations for other world impacted coastal systems.

Area	Cu	Zn	Pb	Ni	Mn	Cd	Reference
Izmit Bay, Turkey	89.4	754	94.9	52.1	–	6.3	[56]
Ribeira Bay, Brazil	24.6	109	22.9	47	466	0.207	[59]
Sepetiba Bay, Brazil	31.9	567	40	22.3	595	3.22	[59]
Mejillones Bay,Chile	–	29.7	–	20.6	93.8	21.9	[58]
Algeciras Bay, Spain	17	73	24	65	534	0.3	[60]
Taranto Gulf, Italy	47.4	102.3	57.8	53.3	893	–	[11]
Tivoli South Bay,USA	17.6	92.8	26.3	–	–	–	[61]
Bay of Bengal, India	677.7	60.39	25.66	34.03	366.66	5.24	[57]
Gulf of Mannar, India	57	73	16	24	305	0.16	[62]
Gulf of Naples, Italy	27.2	602	221	6.93	1550	0.57	[63]
Hong Kong, China	118.68	147.73	53.56	24.72	523.99	0.33	[64]
This study	74.11	689.39	123.98	43.47	750.64	26.81	

Note: “–”  =  no data.

### Correlation between Heavy Metals

Correlation analyses have been widely applied in environmental studies. They provided an effective way to reveal the relationships between multiple variables in order to understand the factors as well as sources of chemical components [50], [65]. Heavy metals in environment usually have complicated relationships among them. The high correlations between heavy metals may reflect that the accumulation concentrations of these heavy metals came from similar pollution sources [66], [67]. Results of Pearson’s correlation coefficients and their significance levels (P<0.01) of correlation analysis were shown in Table 5. The concentrations of Cu, Zn, Pb, Ni and Cd showed strong positive relationship (P<0.01) with each other. This shows that Cu, Zn, Pb, Ni and Cd come from the same source. However, the concentration of Mn showed very weak correlations with the concentrations of the other metals, except Ni. This indicates that Mn have different sources than Cu, Zn, Pb and Cd. Han et al [68] also found the similar result about Mn by multivariate analysis.

**Table 5:** Correlations between heavy metal concentrations.

	Cu	Zn	Pb	Ni	Mn	Cd
Cu	1.000					
Zn	0.919**	1.000				
Pb	0.870**	0.824**	1.000			
Ni	0.499**	0.524**	0.472**	1.000		
Mn	0.115	0.270	0.238	0.515**	1.000	
Cd	0.906**	0.885**	0.972**	0.488**	0.285	1.000

Levels of significance:

* P<0.05;

** P<0.01.

### Spatial Distribution of Heavy Metals in the Sediments of Jinzhou Bay

Geostatistics is increasingly used to model the spatial variability of contaminant concentrations and map them using generalized least-squares regression, known as kriging [69]–[71]. The probability map produced based on kriging interpolation and kriging standard deviation integrates information about the location of the pollutant source and transport process into the spatial mapping of contaminants [71], [72]. There are a lot of studies of the performance of the spatial interpolation methods, but the results are not clear-cut [73]. Some of them found that the kriging method performed better than inverse distance weighting (IDW) [74]; while others showed that kriging was no better than alternative methods [75]. For example, Kazemi and Hosseini [76] compared the ordinary kriging (OK) and other three spatial interpolation methods for estimating heavy metals in sediments of Caspian Sea, they found that the OK realization smoothed out spatial variability and extreme measured values between the range of observed minimum and maximum values for all of the contaminants.

The spatial distribution of metal concentrations is a useful tool to assess the possible sources of enrichment and to identify hot-spot area with high metal concentrations [48], [49]. Semivariogram calculation was conducted and experimental semivariogram of sediment heavy metal concentrations could be fitted with the Gaussian model for Cu, Zn, Pb, Cd, Ni and Mn. The theoretical variation function and experimental variation function exhibits a better fitting (Table 6). The values of R were significant at the 0.01 level by F test, which shows that the semivariogram models well reflect the spatial structural characteristics of sediemnt heavy metals.

**Table 6:** Parameters and F-test of fitted semivariogram models (Gaussian model) for heavy metals in sediment.

	Nugget (C_0_)	Sill (C_0_+C)	C/(C_0_+C)	Range	R^2^	RSS	F test
Cu	5200	111500	0.953	0.0744	0.833	1.41E+09	24.94**
Zn	170000	4450000	0.962	0.0831	0.868	1.38E+12	32.88**
Pb	7900	106900	0.926	0.0883	0.837	7.23E+08	25.67**
Ni	10	7460	0.999	0.0277	0.513	3.81E+07	5.27**
Mn	7370	38310	0.808	0.0710	0.946	2.71 E+07	87.59**
Cd	220	5550	0.960	0.0935	0.921	8.49 E+05	58.29**
RI	890000	22880000	0.961	0.1004	0.927	1.12E+13	63.49**

** Significance at α = 0.01 level of F test.

**Figure 2:**
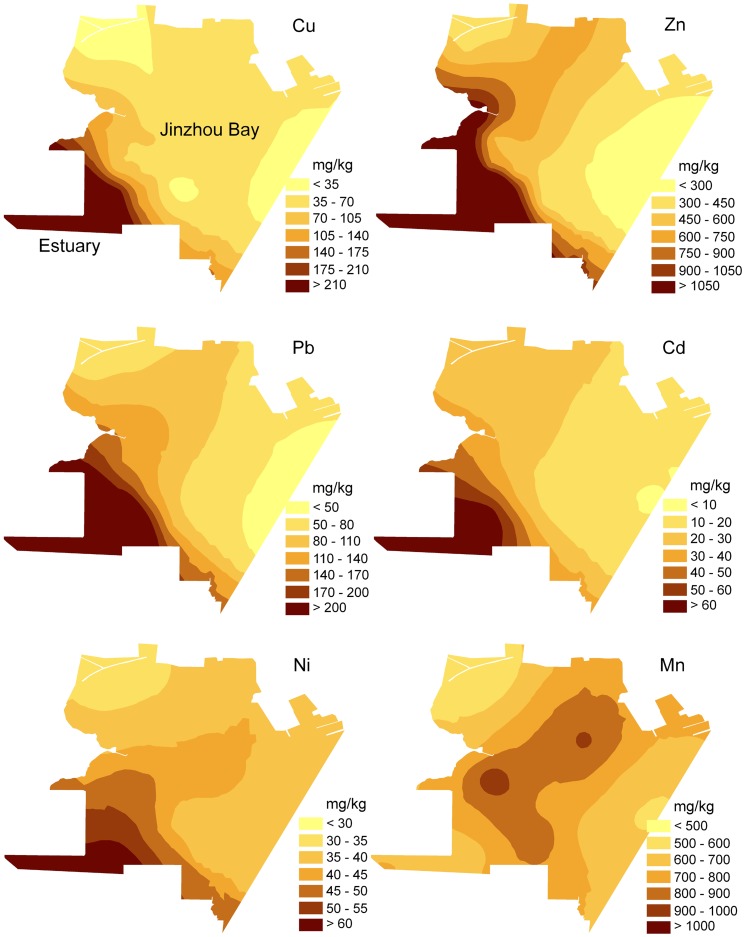
Estimated concentration maps for Cu, Zn, Pb, Cd, Ni and Mn (mg/kg).

The estimated maps of Cu, Zn, Pb, Cd, Ni and Mn clearly identified that the river, where the Huludao Zinc Smelter (HZS) is located at, is the most important source of heavy metals except Mn (Fig. 2). Among these metals, Cu, Zn, Pb and Cd showed a very similar spatial pattern, with contamination hotspots located at the estuary area, and their concentrations decreased sharply with the distance farther away from the estuary, indicating that they were from the same sources. The concentration of Ni also showed a similar pattern with the concentrations of Cu, Zn, Pb and Cd, but it changed not as sharply as the latter one. However, the concentration of Mn showed a completely different pattern with the others, indicating the industrial activities are not the source of Mn in the Jinzhou bay, and it may be related to geological factor. The mineral constituents of Jinzhou bay consist mainly of hornblende, epidote and magnetite. The percentage of hornblende, which is rich in Mn element, varied from 24.78% up to 64.70% [77]. This confirms that the concentration of Mn comes from the geological sources. The feature of point sources lies in that the inputs of heavy metals occur over a finite period of time and may have been effectively retained in the sediments near the sources, rather than re-suspended and distributed uniformly throughout the region [66]. Distinct from point sources, metals from non-point sources are more uniformly distributed throughout the area [50]. The results of this study closely correspond to this association.

The Spatial pattern of heavy metal in Jinzhou bay also provided a refinement and reconfirmation of the results in the statistical analysis, in which strong associations were found among Cu, Zn, Pb, Cd and Ni and very weak relations were found between Mn and the other heavy metals except Ni.

### Assessment of Potential Ecological Risk, RI

**Figure 3:**
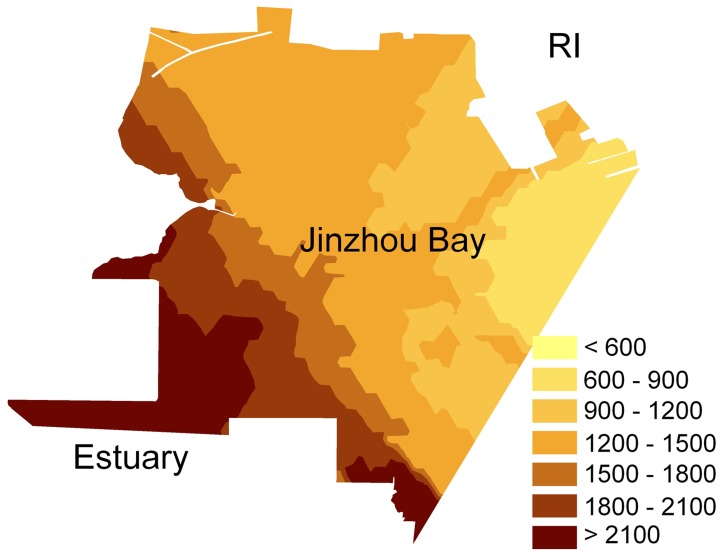
The spatial distribution pattern of RI of sediments in Jinzhou Bay.

Almost all the RI values of sampling sites were higher than 600 except the two samples from Wuli river, indicating that the sediments in the rivers of Huludao city and their estuary and Jinzhou bay exhibited very high ecological risk of heavy metals (Fig. 3, Table 7). The RI of sediments in the estuary was as high as 34.6 times of the line value for very high ecological risk level, suggesting that the sediments in the estuary were extremely polluted by heavy metals because of industrial discharge. Cd showed the highest potential ecological risk in the heavy metals, which contributed more than 95% of RI in the sampled sediments.

**Table 7:** The heavy metal potential ecological risk indexes in sediments.

Sediments	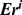						Pollution degree
	Cu	Zn	Pb	Ni	Cd	RI	
Lianshan River	16.64	4.23	9.36	31.89	3190.804	3252.96	very high
Wuli River	7.23	1.71	4.80	17.50	667.43	698.69	very high
Estuary	162.50	41.10	135.54	34.54	20414.20	20787.87	very high
Jinzhou Bay	10.59	4.60	10.33	24.15	1608.68	1658.34	very high

### Temporal Distribution of Heavy Metals in the Sediments of Jinzhou Bay

**Figure 4:**
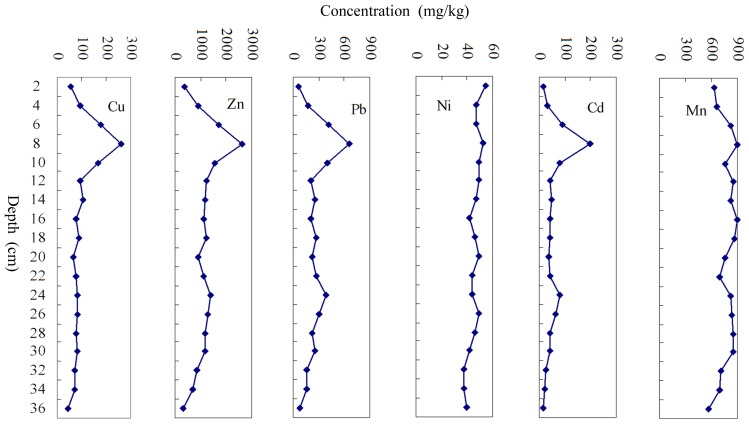
Vertical profiles of heavy metals for sediment core of Jinzhou bay, NE coast of China.

Sediment cores can be used to study the pollution history of aquatic ecosystem [44], [48]. Vertical distribution (0–36 cm) of heavy metals in Jinzhou Bay indicate that the concentration of Cu, Zn, Pb and Cd show similar vertical patterns (Fig. 4). The values of Cu, Zn, Pb and Cd increased sharply from the surface to its highest concentration at the depth of 8 cm and then decreased rapidly at a depth of 20 cm. The values of Cu, Zn, Pb and Cd varied slightly from a depth of 21–36 cm. The concentration of Ni in the sediment core decreased gradually from the surface with small fluctuations while the Mn remained relatively consent throughout the core.

According to the sedimentation rate of about 1.0 cm/yr [78], the bottom of the sediment core (at the depth of 36 cm) was polluted about in 1973, 36 years after the set up of Huludao Zinc Smelter (HZS) in 1937. The heavy metals concentrations were already greatly higher than the national guideline values of marine sediment quality of China (GB 18668-2002), especially the concentration of Cd as high as 31 times of the latter. Related to annual Zn yields of HZS between 1973 and 2010 with the concentrations of Cu, Zn, Pb and Cd in sediment core, they showed similar temporal patterns, especially around the year 2000, the peak of Zn yield was followed the highest heavy metal concentrations at the depth of 8 cm of sediment core. This is conclusive evidence that Zn smelting operation was the dominant pollution source of aquatic environment in Jinzhou Bay. With the disposal and reuse of heavy metal wastewater from HZS around 2000, although the Zn yields increased year by year, the sediment pollution was alleviated gradually since 2000. The concentration of Ni in the sediment core decreased gradually from the surface with small fluctuations, the value varied from 54.36 mg/kg at the surface to 37.09 mg/kg at the depth of 32 cm. This also clearly indicated that the content of Ni in the sediment was come from the industrial discharges. Vertical profile of Mn shows some fluctuations in its concentration, and no obvious correlation with the depth of sediment core was observed. This indicates that the concentration of Mn is not under the control of human factors, as the result showed by the spatial pattern of Mn.

### Conclusion

This study investigated the concentrations of heavy metals in the sediments from urban-stream, estuary and Jinzhou bay of the coastal industrial city, NE China. The results showed the impact of anthropogenic agents on abundances of heavy metals in sediments. The sediments are found to be extremely contaminated due to many years of random dumping of hazardous waste and free discharge of effluents by industries like Huludao Zinc Smelter (the largest zinc smelting plant in Asia), the Jinxi oil refinery and Jinhua chemical engineering, Huludao’s massive shipyard and several arms factories. The potential ecological risk of sediments in lower river reaches, estuary and Jinzhou bay is at very high level, and Cd contributed more than 95% of RI in the sampled sediments. The estuary is the most polluted area, and its RI value was as high as 34.6 times of the line value for very high ecological risk level. The closer the distance to the estuary is, the higher RI values of sediments in Jinzhou bay are. The results of this research updated the information for effective environmental management in the industrial region. This study clearly highlights the urgent need to make great efforts to control the industrial discharges in the coastal area, and the immediate measures should be carried out to minimize the contaminations, and to prevent future pollution problems.
